# Phenotypes and the Importance of Genetic Analysis in Adult Patients with Nephrolithiasis and/or Nephrocalcinosis: A Single-Center Experience

**DOI:** 10.3390/genes16050501

**Published:** 2025-04-27

**Authors:** Elena Emanuela Rusu, Bogdan Marian Sorohan, Robert Pandele, Andreea Popescu, Raluca Bobeica, Sonia Balanica, Diana Silvia Zilisteanu, Alexandru Iordache, Adrian Lungu, Gener Ismail

**Affiliations:** 1 Department of Nephrology, “Carol Davila” University of Medicine and Pharmacy, 020021 Bucharest, Romania; bogdan.sorohan@yahoo.com (B.M.S.); gabriel-robert.pandele@rez.umfcd.ro (R.P.); andreea-lidia.blaga@rez.umfcd.ro (A.P.); diana.zilisteanu@umfcd.ro (D.S.Z.); gener.ismail@umfcd.ro (G.I.); 2 Department of Nephrology, Fundeni Clinical Institute, 022328 Bucharest, Romania; ralbobeica@gmail.com (R.B.); sonia.balanica@gmail.com (S.B.); 3 Department of Uronephrology and Kidney Transplantation, Fundeni Clinical Institute, 022328 Bucharest, Romania; 4 Department of Urology, Fundeni Clinical Institute, 022328 Bucharest, Romania; iordachemd@gmail.com; 5 Department of Pediatric Nephrology, Fundeni Clinical Institute, 022328 Bucharest, Romania; adilungu@mediakompass.ro

**Keywords:** nephrolithiasis, nephrocalcinosis, genetic testing, hereditary etiology, Mendelian, phenotype, genotype, molecular analysis

## Abstract

Background: Molecular analysis in patients with nephrolithiasis (NL) and/or nephrocalcinosis (NC) enables more accurate evaluation of underlying etiologies. The existing clinical evidence regarding genetic testing in adults with NL comprises only a few cohort studies. Materials and Methods: We retrospectively analyzed 49 adult patients diagnosed with NL and/or NC from a single center, on whom we performed a genetic test using a nephrolithiasis panel. We reviewed the phenotype of the patients and compared the cases with positive and negative molecular diagnosis. Results: In total, 49 adult patients with NL and/or NC underwent genetic testing. Of the tested patients, 29 (59.2%) patients had 24 abnormal variants in 14 genes. Mendelian diseases were diagnosed in 14 (28.6%) cases: cystinuria (*SLC3A1, SLC7A9*; *n* = 4), hereditary distal renal tubular acidosis (*SLC4A1*; *n* = 3), Dent disease (*CLCN5*; *n* = 2), familial hypomagnesaemia with hypercalciuria and nephrocalcinosis (*CLDN16*; *n* = 1), infantile hypercalcemia type 1 (*CYP24A1*; *n* = 1), primary hyperoxaluria type 1 (*AGXT*; *n* = 1), Bartter syndrome type 2 (*KCNJ1*; *n* = 1), and autosomal dominant tubulointerstitial kidney disease (*UMOD*; *n* = 1). Eight (16.3%) patients had pathogenic or likely pathogenic monoallelic variants as predisposing factors for NL and/or NC, and seven (14.3%) had biallelic or monoallelic variants of uncertain significance. Patients with positive genetic tests had a lower estimated glomerular filtration rate (*p* = 0.03) and more frequent NL associated with NC (*p* = 0.007) and were unlikely to have arterial hypertension (*p* = 0.03) when compared with patients with negative tests. Conclusions: Our study shows an increased effectiveness of molecular diagnosis and highlights the benefits of genetic testing. NL associated with NC and the presence of chronic kidney disease are the characteristics that should prompt the clinician to suspect an inherited form of NL and/or NC.

## 1. Introduction

Nephrolithiasis (NL) is a common disease, with a globally increasing prevalence to more than 10% [1,2] and with a recurrence rate ranging from 6.1% to even 82.4% in patients with specific mineral composition of the stones [3,4]. Nephrolithiasis and its parenchymal form, nephrocalcinosis (NC), are associated with significant morbidity and progression to chronic kidney disease [5,6]. NL has a complex etiology, with dietary, environmental, and genetic components. A positive family history of NL increases the risk of an earlier onset of stone formation and frequent recurrences [7,8]. Twin studies underline the heritability of NL, showing 57% heritability among men and 46% heritability among women [9]. The evidence of genetic associations with NL was also underscored by genome-wide association studies (GWAS) [5,6]. The most recent GWAS meta-analysis for kidney stone disease identified 44 susceptibility loci [5]. Among these, the lead variants at 12 loci were associated with the calcium, phosphate, or 25-hydroxyvitamin D concentration in the serum [5]. As of now, there are more than 40 known genes that have been shown to cause Mendelian (known also as “monogenic”) NL and/or NC [10]. Molecular analysis in patients with NL and/or NC enables appropriate evaluation. Genetic testing for known pathogenic mutations using gene panels or whole-genome sequencing has become more accessible due to technological advances and cost decreases [11]. European and American Urological guidelines underline the factors and the diseases associated with a high risk of stone formation, including genetic diseases, but, currently, there are no clear recommendations to guide physicians for genetics referral [12,13]. Several clinical and laboratory indices have been described to prompt clinicians to perform comprehensive investigations to identify the genetic substrate of NL [14,15]. Thus, a full assessment of the clinical phenotype could offer significant data for the suspicion of genetic nephrolithiasis, leading to genetic screening. The detection of genetic disease will allow for the elucidation of the pathophysiological mechanism of the kidney stones and the implementation of personalized management, especially for certain genetic etiologies of NL that benefit from new therapeutic options [11,16].

The existing clinical evidence regarding genetic testing in adults with kidney stone disease comprises only a few cohort studies [17,18,19,20,21,22]. Numerous hereditary stone conditions lead to NL and/or NC, such as cystinuria, distal renal tubular acidosis (dRTA), Dent disease, familial hypomagnesaemia with hypercalciuria and nephrocalcinosis (FHHNC), infantile hypercalcemia type 1 (HCINF1), and primary hyperoxaluria type 1 (PH1) [14,15,16,17,18,19]. The most common genetic diagnosis in clinical studies is cystinuria [17,19]. Kidney failure has been frequently identified in patients with hereditary NL and/or NC; therefore, their prompt identification has important prognostic and therapeutic implications [14,17].

Our single center study reports the genetic and clinical characteristics of patients with NL and/or NC, on whom we performed genetic testing.

## 2. Materials and Methods

### 2.1. Study Cohort

We report a retrospective cohort observational study that included adult patients with a diagnosis of nephrolithiasis and/or nephrocalcinosis, on whom we performed a genetic test for the suspicion of hereditary etiology of NL/NC, between September 2020 and May 2024. The study was conducted in the Nephrology Department, Expert Center for Rare Diseases of Reno-Urinary System, Fundeni Clinical Institute. The study was conducted in accordance with the principles of the Helsinki Declaration and approved by the Institutional Ethics Committee of the Fundeni Clinical Institute (No. 66359/2024). The patients signed an informed consent form for participation in the clinical research at hospital admission. Informed consent for genetic studies was obtained from each patient.

The inclusion criteria are as follows:

- Age ≥ 18 years;
- Kidney stone disease: NL and/or NC;
- Onset of kidney stone disease before 40 years of age;
- The presence of at least one of the following: family history of NL and/or NC, indicative phenotype (such as distal renal tubular acidosis, particular stone composition), recurrent NL, and chronic kidney disease (CKD).

Exclusion criteria:

- Patients diagnosed with a known non-genetic cause of kidney-stone-related disease.

### 2.2. Clinical Assessments

All of our patients were diagnosed with NL and/or NC through renal ultrasound or computed tomography. Demographics, relevant personal and family pathological history, appropriate chemistry, urinary and stone analyses results, metabolic evaluation, comorbidities, past and current medical treatment, and former urological interventions were recorded. The estimated glomerular filtration rate (eGFR) was calculated using the Chronic Kidney Disease Epidemiology Collaboration (CKD-EPI) equation for adults. Chronic kidney disease was classified based on the GFR categories as follows: G1 for eGFR ≥ 90 mL/min/1.73 m^2^; G2 for eGFR 60–89 mL/min/1.73 m^2^; G3a for eGFR 45–59 mL/min/1.73 m^2^; G3b for eGFR 44–30 mL/min/1.73 m^2^, G4 for eGFR 15–29 mL/min/1.73 m^2^; and G5 for eGFR < 15 mL/min/1.73 m^2^ (end-stage kidney disease—ESKD) [23].

### 2.3. Genetic Testing

The blood samples were analyzed at one of the following genetic testing laboratories: Blueprint Genetics (Helsinki, Finland) or Invitae (San Francisco, CA, USA). The nephrolithiasis panel was customized by the laboratory based on the scientific literature, mutation databases, and laboratory experience. We used the nephrolithiasis panel, which includes 45 genes at Blueprint Genetics [24] and 40 genes at Invitae [25], but the requesting physician could add different genes according to clinical suspicion.

The following genes were examined:

- Genes related to calcium metabolism: *ADCY10*, *CASR*, *CLCN5*, *CLDN16*, *CLDN19*, *CYP24A1*, *GNA11*, *HNF4A*, *OCRL*, *VDR*;
- Genes related to renal tubular acidosis: *ATP6V0A4*, *ATP6V1B1*, *SLC4A1*, *CA2*, *FOXI1*;
- Genes related to phosphate metabolism: *SLC9A3R1*, *ALPL*, *SLC34A1*, *SLC34A3*;
- Genes related to uric acid metabolism: *UMOD*, *HPRT1*, *PRPS1*, *SLC22A12*, *SLC2A9*;
- Bartter syndrome genes: *BSND*, *CLCNKA*, *CLCNKB*, *KCNJ1*, *MAGED2*, *SLC12A1*;
- Cystinuria genes: *SLC3A1*, *SLC7A9*;
- Genes related to oxalate metabolism: *AGXT*, *GRHPR*, *HOGA1*, *SLC26A1*;
- Other genes: genes related to xanthinuria (*XDH* and *MOCOS* genes), Molybdenum cofactor deficiency (*MOCS1* and *MOCS2* genes), Molybdenum cofactor deficiency C (*GPHN* gene), adenine phosphoribosyltransferase deficiency (*APRT* gene), and 3-methylglutaconic aciduria type VIIB (*CLPB* gene).

The nephrolithiasis panel was sectioned from the clinical-grade next-generation sequencing (NGS) assay [24,25]. Variant classification followed the American College of Medical Genetics and Genomics (ACMG) guideline 2015 [26]. Sequence and copy number variants classified as pathogenic (P), likely pathogenic (LP), and variants of uncertain significance (VUS) were confirmed by the laboratory using bi-directional Sanger sequencing or orthogonal methods [24,25].

### 2.4. Study Endpoints

Our study endpoints were as follows:

- To review the genotype and phenotype of hereditary NL and/or NC in adult patients from a single center;
- To compare the clinical and biological characteristics of patients with positive and negative genetic diagnosis;
- To evaluate the clinical utility of molecular genetic diagnosis.

### 2.5. Statistical Analysis

Data were presented as frequencies with percentages for categorical variables, as means with standard deviation for continuous parametric data, and as medians with interquartile ranges for continuous nonparametric data. For variable comparison, chi-square or Fisher’s exact tests were used as appropriate for categorical data, Student’s t-test for continuous parametric data, and the Mann–Whitney U test for continuous nonparametric data. Paired-samples t-tests were used to evaluate the mean difference of a variable between baseline and the last time of follow-up, where the baseline was defined as the time of kidney biopsy. A *p* value < 0.05 was considered statistically significant.

Statistical analysis was performed and figures were created using SPSS version 26 (SPSS Inc., Chicago, IL, USA), STATA version 14 (StataCorp, College Station, TX, USA), and GraphPad Prism version 9.3.1 (1992–2021 GraphPad Software, LLC, Boston, MA, USA).

## 3. Results

### 3.1. Study Cohort and Genetic Analysis

Our single-center study included 49 adult patients with NL and/or NC (23 males and 26 females), with a mean age at the time of recruitment 35.1 ± 10.2 years (ranging from 18 to 58 years), on whom we performed genetic testing using a nephrolithiasis panel. The great majority of patients were from unrelated families of Caucasian origin (only two families with two members per family), and only one patient had African ethnicity. We did not record any case of consanguinity in our patients. Among the 49 adult patients included in the analysis, 37 (75.5%) patients had isolated NL, 3 (6.1%) had isolated NC, and 9 (18.4%) patients had combined NL and NC.

**Figure 1:**
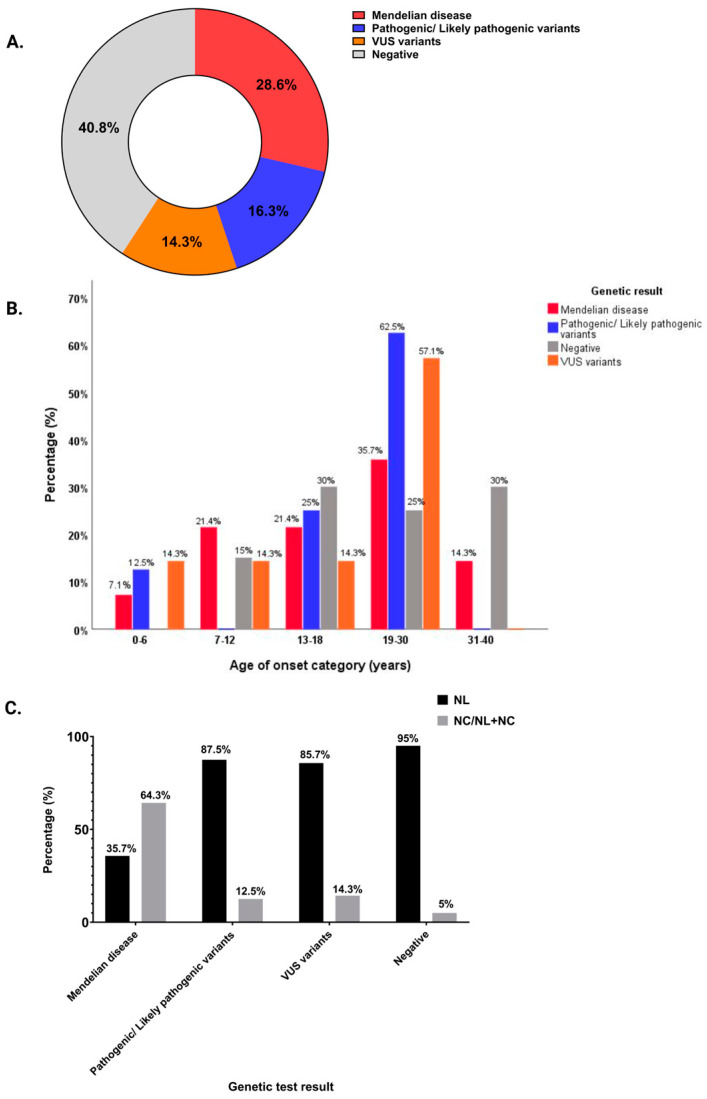
Molecular diagnosis results in patients with NL/NC. (**A**) Diagnostic yield of patients with NL/NC: 28.6% Mendelian disease, 16.3% P/LP variants predisposing to NL/NC, 14.3% VUS (including biallelic and monoallelic VUS), 40.8% negative. (**B**) Percentage of patients with positive or negative molecular diagnosis according to the age of the onset of the disease. (**C**) A summary of the distribution of NL only, or isolated NC/NL in association with NC, according to the result of the genetic test. NL, nephrolithiasis; NC, nephrocalcinosis; NL + NC, nephrolithiasis plus nephrocalcinosis; VUS, variants of uncertain significance.

In 59.2% (29/49) of patients, we found 24 abnormal variants in 14 genes, while 40.8% (20/49) had a negative genetic test (Table 1). Mendelian kidney stone disease was diagnosed in 28.6% (14/49) of patients, P/LP monoallelic variants as risk factors for NL/NC were determined in 16.3% (8/49) of patients, and 14.3% (7/49) of patients had biallelic or monoallelic VUS (Figure 1A).

We diagnosed a Mendelian form of NL/NC in 14 patients, in whom we identified pathogenic or likely pathogenic variants of mutations that align with the classical inheritance mode of NL/NC (Table 2).

In addition, we found a possible genetic diagnosis for the patients who had positive genetic tests with VUS that aligns with the inheritance mode of the Mendelian form of NL/NC. In our cohort, two patients had VUS that suggested a possible molecular diagnosis (Table 3), reported in the VUS group. In the case of eight patients in whom pathogenic or likely pathogenic monoallelic variants were detected and who did not meet the criteria for the inheritance mode of a Mendelian form of NL/NC, we considered that the identified mutations were predisposing factors for kidney stone disease (Table 4). There were also five patients with monoallelic VUS of known NL/NC genes (Table 5).

We compared the clinical and laboratory characteristics of the patients in whom we identified at least one genetic abnormality (positive genetic test) with the patients with negative genetic tests (Table 1). Among patients with a positive genetic test, nine patients (31%) presented with NL associated with NC, versus none within the patients with a negative genetic test (*p* = 0.007). Also, the patients with a positive genetic test presented a statistically significant lower eGFR (*p* = 0.03) and a more severe stage of chronic kidney disease when compared with the patients with a negative genetic test. Patients with a negative genetic test had hypertension more frequently (*p* = 0.03). Characteristics potentially related to hereditary NL/NC, such as the onset age, positive family history, recurrent stones, and bilateral stones, were not significantly different among the patients with a positive versus negative genetic test. In addition, there were no significant differences regarding blood parameters and history of surgical interventions between the two groups.

We analyzed the cohort according to the age at the first stone event. Splitting the cohort into five age groups, all of the patients with onset of disease between 0 and 6 years had a positive genetic test (Figure 1B). Of note, among the 12 (24.5%) patients with isolated nephrocalcinosis or nephrocalcinosis associated with nephrolithiasis, we found a Mendelian disease in nine patients, a P/LP variant in one patient, VUS in one patient, and a negative test in one case (Figure 1C).

**Figure 2:**
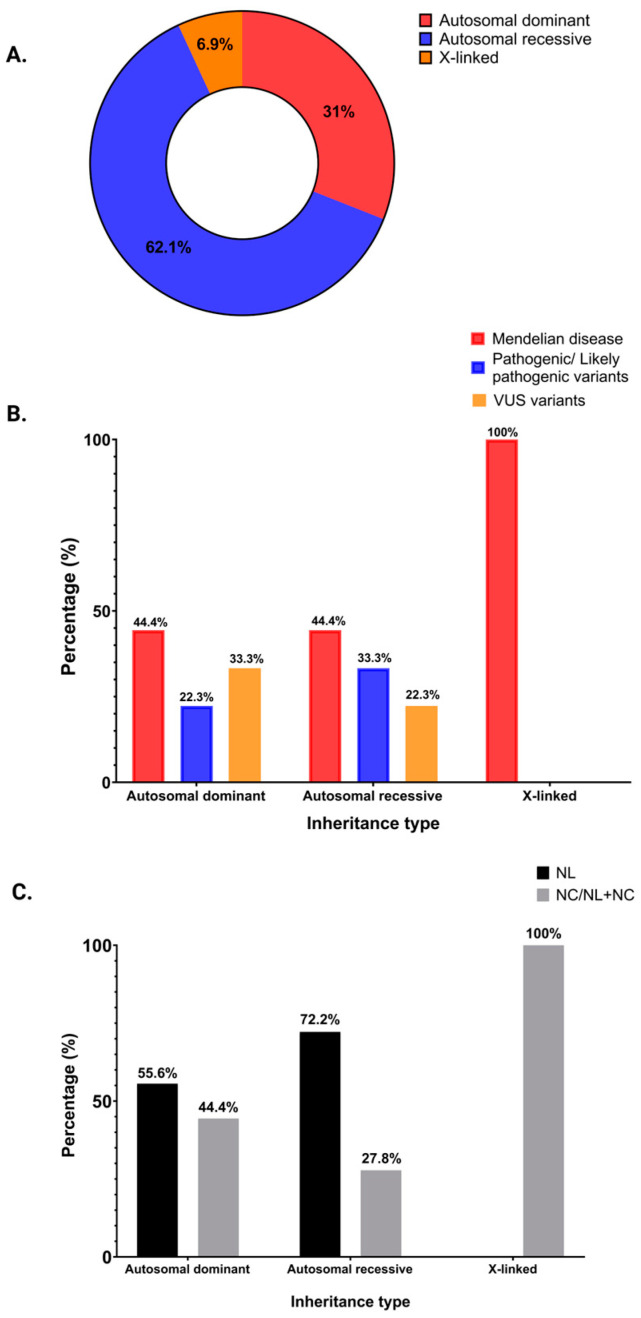
Distribution of inheritance type in patients with positive genetic test. (**A**) Inheritance type in the group of patients with positive genetic tests. (**B**) Inheritance type according to variant classification. (**C**) Distribution of inheritance type for adults with NL, NC/NL plus NC.

The distribution of inheritance type showed that in 18 (62.1%) of the patients with a positive genetic test, we found variants in genes related to an autosomal recessive genetic disease. In nine (31%), we found variants in genes related to an autosomal dominant genetic type of inheritance, and in two (6.9%) patients, we found variants in genes related to an X-linked disease (Figure 2A). Both X-linked cases are Mendelian diseases. However, we did not find a correlation between the inheritance type and the identified mutations (Figure 2B). The patients with the autosomal dominant or recessive type of inheritance had NL and/or NC, while patients with an X-linked disease (Dent’s disease) had isolated NC or NL plus NC (Figure 2C).

Analysis of the allelic state of the detected mutations in positive patients showed that 53.6% of cases had heterozygous mutations, 21.4% of cases had compound heterozygous mutations, 10.7% of cases had homozygous mutations, 10.7% of cases presented mutations for two different genes, and 3.6% of cases had hemizygous mutations (Table 2, Table 3, Table 4 and Table 5).

### 3.2. Genotype and Phenotype of the Positive Patients

#### 3.2.1. Mendelian Forms of NL/NC

Among the 49 patients included in our study, 14 patients (28.6%) were identified with a Mendelian form of NL/NC (Table 2). Deleterious variants were identified in the following genes (*n* = number of patients): *SLC4A1* (*n* = 3), *SLC3A1* (*n* = 2), *SLC7A9* (*n* = 2), *CLCN5* (*n* = 2), *CLDN16* (*n* = 1), *CYP24A1* (*n* = 1), *AGXT* (*n* = 1), *KCNJ1* (*n* = 1), and *UMOD* (*n* = 1). A summary of the clinical, biological, and genetic characteristics of each patient and a short definition of the Mendelian diseases are presented below.

##### Cystinuria

**Figure 3:**
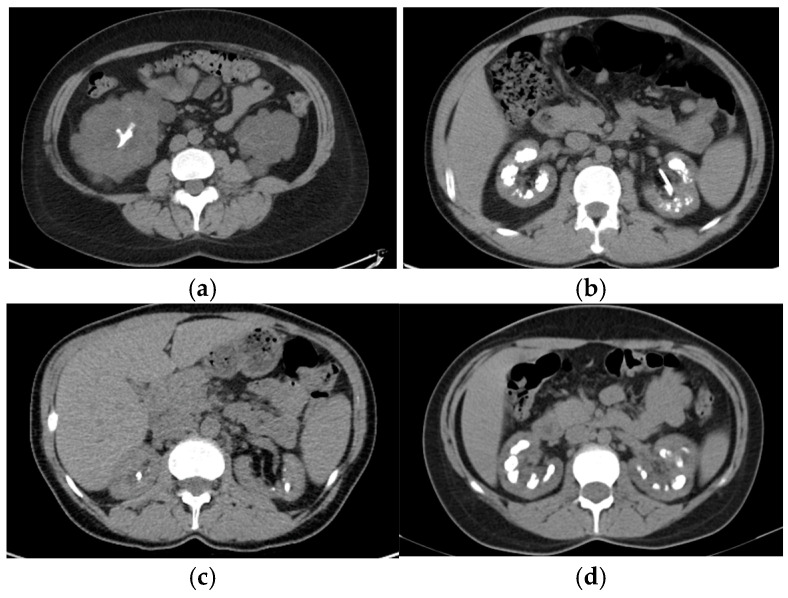
Renal imaging from 4 patients. (**a**) CT scan of the 43-year-old patient with cystinuria and autosomal dominant polycystic kidney disease, transversal section: staghorn calculus in the right kidney; (**b**) CT scan of the 39-year-old patient with hereditary dRTA, transversal section: non-obstructive multiple bilateral kidney stones and nephrocalcinosis, left ureteral JJ stent; (**c**) CT scan of the 42-year-old patient with PH1, transversal section: non-obstructive bilateral kidney stones and intraparenchymatous calcifications; (**d**) CT scan of the 32-year-old patient with Bartter syndrome type 2, transversal section: bilateral medullary nephrocalcinosis.

In our cohort, cystinuria was the most common Mendelian diagnosis (*n* = 4). We detected biallelic pathogenic variants in *SLC3A1* for two patients (type A cystinuria) and biallelic pathogenic and likely pathogenic variants in *SLC7A9* for another two patients (type B cystinuria). All cystinuria patients presented with CKD, two of them even having ESKD. The first patient, a 45-year-old man, had compound heterozygous pathogenic variants in *SLC3A1*. He was diagnosed with cystine NL at the age of 35 years and had recurrent stones that required multiple urologic interventions. The kidney function slowly decreased, reaching ESKD at 42. The second patient, a 43-year-old female, had her first cystine stone at 25. The genetic test showed homozygous pathogenic variants in *SLC3A1*. She also presented with an associated LP mutation in the *PKD1* gene. The computed tomography exam showed staghorn NL in her right kidney, along with multiple cysts in both kidneys (Figure 3a). The two patients with type B cystinuria carried compound heterozygous mutations in the *SLC7A9* gene. A 53-year-old male had his first stone at 14, and then he had recurrent cystine NL requiring multiple urological interventions. He had now reached CKD G4. He had two mutations of the *SLC7A9* gene, c.313G>A, p.Gly105Arg; c.690G>A, p.Trp230*, the second one being a novel variant. The other patient was a 52-year-old woman with the onset of cystine NL at the age of 29, recurrent NL, and progressive CKD that required hemodialysis when she was 48 years old.

##### Hereditary Distal Renal Tubular Acidosis

In our study, eight patients had a clinical suspicion of genetic dRTA. Molecular analysis revealed known pathogenic variants of SLC4A1 in three cases. A 27-year-old woman with a history of recurrent carbapatite NL, with the first stone at the age of 18 years, bilateral medullary NC, presented with dRTA and CKD G3a. The patient could not provide data regarding family history. Another patient, a 39-year-old man, presented with CKD G3a, NC, recurrent carbapatite stones, with NL onset at 12, and multiple urological interventions. An image from the computed tomography (CT) scan is presented in Figure 3b. We genetically investigated his child and his brother, and they had the same pathogenic mutation. The brother, 35 years old, had carbapatite NL, NC, dRTA, CKD G3b, with the onset of hypokalemia symptoms at 13, when he was diagnosed with dRTA. Also, we evaluated their father clinically and biologically, who presented with nephrolithiasis and dRTA. In this family, we observed adolescent disease onset and the autosomal dominant pattern of inheritance.

##### Dent Disease

In our cohort, two patients had a causative mutation in the *CLCN5* gene. One pathogenic variant was identified in a 53-year-old man with a kidney transplant who had NL, NC, and multiple bilateral cysts in the native kidneys. He was diagnosed with kidney failure at 28 years and started hemodialysis; then, he received a kidney transplant. He had a brother who died before the age of 10 with suspected kidney disease, and he had two other brothers with kidney stone disease and kidney failure. He performed the genetic test after his nephew was diagnosed with kidney stones at the age of 2. Our patient was also found to have VUS c.2622C>G, p.Phe874Leu in the *GANAB* gene, which explained the presence of kidney cysts. In this case, the disease was diagnosed in a late stage of CKD, but performing a pedigree will help other family members to be diagnosed in an earlier stage of the disease. Another pathogenic variant of the *CLCN5* gene was identified in a 27-year-old woman with severe medullary NC and mild proteinuria but normal kidney function.

##### Familial Hypomagnesemia with Hypercalciuria and Nephrocalcinosis

In the present study, homozygous mutations of the CLDN16 gene were detected in a 20-year-old female who was clinically diagnosed with hypomagnesemia, hypocalcemia, hypercalciuria, bilateral nephrocalcinosis, and CKD stage G4. The patient was 7 years old at the first manifestation of the disease but received a molecular diagnosis after a delay of 13 years when she showed advanced kidney disease.

##### Infantile Hypercalcemia Type 1

Our cohort included a 24-year-old male patient who was referred for renal investigations after the accidental finding of increased serum creatinine. He presented with hypercalcemia, hypophosphatemia, suppressed intact parathyroid hormone, low serum alkaline phosphatase, an eGFR of 72 mL/min/1.73 m^2^, and hypercalciuria. The CT scan revealed bilateral nephrocalcinosis and a renal cyst, typical findings for patients with HCINF1. The patient had biallelic mutations in *CYP24A1*, leading to the loss of this enzyme’s function.

##### Primary Hyperoxaluria Type 1

One 42-year-old female with NL, who had her first stone at the age of 6, and who had NC was diagnosed with PH1 (Figure 3c). She was found to have biallelic pathogenic mutations in the *AGXT* gene. After family screening, the mother was found to be positive for the familial frameshift deletion, a null mutation, and the father, also a stone former, was found to be positive for the familial missense mutation, which is sensitive to vitamin B6. The patient presented with severely impaired kidney function, high urinary oxalate, and increased plasma oxalate, but no signs of systemic oxalosis.

##### Bartter Syndrome Type 2

We report the case of a 32-year-old female of African origin, who presented with symptoms of thirst, salt craving, polydipsia, polyuria, and weakness since childhood starting at the age of 12 years. She was previously diagnosed with chronic hypokalemia, tubular acidosis type 3, bilateral NC, diabetes insipidus, hyperreninemic hyperaldosteronism, primary hyperparathyroidism, and parathyroid adenoma. At the age of 31, she presented with obstructive pyelonephritis with acute kidney injury. The analysis of the kidney stone showed a mixed composition with calcium oxalate, calcium phosphate, and struvite. When she was referred to our clinic, she revealed symptoms of polydipsia, polyuria, and fatigue. Laboratory investigations showed increased serum creatinine with an estimated GFR 51 mL/min/1.73 m^2^, hypokalemia, elevated PTH levels, intermittent metabolic alkalosis, and normal blood calcium, sodium, magnesium, and phosphorus concentrations. Urinalysis showed hypercalciuria and hypocitraturia. The computed tomography exam showed bilateral medullary NC (Figure 3d). In our clinic, she was diagnosed with Bartter syndrome type 2, an autosomal recessive renal tubular disorder, based on the presence of homozygous pathogenic mutations in the *KCNJ1* gene.

##### Autosomal Dominant Tubulointerstitial Kidney Disease (ADTKD)

ADTKD—*UMOD* is the most common form of inherited CKD after autosomal dominant polycystic kidney disease, with a prevalence of 1% in CKD G3–G5 and 2% in patients with ESKD [27]. Mutations in the *UMOD* gene have been associated with the risk of progressive CKD and other diseases, such as hypertension and kidney stones [27]. We detected one monoallelic LP variant in the *UMOD* gene in a 34-year-old female with CKD stage G3a, bland urinalysis, sporadic kidney stones, and a familial history of CKD. She was diagnosed with CKD at the age of 25 when a kidney biopsy was performed, but the histology was not conclusive for the clinical diagnosis. After 9 years, she was finally diagnosed with ADTKD following the genetic test.

#### 3.2.2. Patients with a Possible Genetic Diagnosis for NL/NC

Two patients presented variants of unknown significance with respect to the relevant mode of inheritance and showed a possible genetic diagnosis (Table 3). The first patient, a 31-year-old woman, presented a heterozygous mutation in the *ADCY10* gene, c.4558G>A, p.Val1520Ile, with an autosomal dominant inheritance pattern, which was classified as VUS. She was admitted to our clinic for renal colic and urinary tract infection. Clinical evaluation revealed NL with onset at the age of 15, calcium oxalate monohydrate stone composition, normal kidney function, and hypercalciuria. The family history showed the diagnosis of kidney stone disease in her father’s father, her father, and her 6-year-old daughter. *ADCY10* gene mutations are associated with autosomal dominant familial idiopathic hypercalciuria. This is a new variant that was not reported in the literature in individuals affected with *ADCY10*-related disease. The second patient, a 40-year-old man, had biallelic VUS in the *SLC34A3* gene, which encodes the sodium–phosphate co-transporter proteins 2c. Starting when he was 37 years old, he presented with NL with multiple kidney stones and urologic interventions. Stone analysis showed mixed composition: calcium carbonate, calcium phosphate, and struvite. The patient had a decreased serum phosphate level, normal levels of PTH, and normal kidney function. Analysis of the 24-h urine showed hypercalciuria and normal phosphaturia. He has a family history of NL, with both of his parents having episodes of NL with onset at adult age.

#### 3.2.3. P/LP Monoallelic Variants Predisposing to NL/NC

In our cohort, eight patients were identified with a genetic predisposition to NL/NC due to the detection of monoallelic P/LP variants in known genetic NL/NC genes (Table 4). Remarkably, two of these patients (two sisters) had two different P/LP monoallelic variants predisposing them to NL/NC. The most frequent predisposition was for cystinuria and renal phosphate wasting.

We identified two patients with monoallelic P/LP variants for the *SLC3A1* gene and two patients for the *SLC7A9* gene. A 32-year-old female heterozygous for the *SLC3A1* variant c.1400T>C, p.Met437Thr presented with multiple bilateral stones, many episodes of renal colic, and multiple urological interventions. The second pathogenic monoallelic mutation in the *SLC3A1* gene was detected in a 30-year-old male with NL. Two young adults (a 26-year-old man and a 30-year-old woman) with calcium-oxalate NL had monoallelic mutations in the *SLC7A9* gene. All four patients presented with calcium-based stones and preserved kidney function.

Our cohort also includes two female patients who are sisters. Both had an interesting biallelic heterozygous pattern that contributed to their clinical phenotype: a monoallelic *AGXT* variant c.107G>A, p.Arg36His, and a monoallelic *SLC34A3* variant, c.575C>T, p.Ser192Leu. The older sister, the 28-year-old, had bilateral calcium oxalate monohydrate kidney stones, repeated episodes of renal colic starting from the age of 18, and CKD G2 (eGFR = 63 mL/min/1.73 m^2^). She presented with normal calcium, phosphorus, and iPTH. The 25-hydroxy-vitamin D was decreased, and 1,25-dihydroxy-vitamin D was normal. The urine metabolic evaluation showed mildly increased urinary oxalate and decreased urinary phosphorus. She had normal values for calcinuria and citraturia. The younger sister, the 25-year-old, had NL with onset at the age of 20 and mildly decreased eGFR. Urinary metabolic evaluation showed a mildly increased urinary oxalate. The combined effect of the monoallelic *AGXT* and *SLC34A3* mutations can influence the clinical course.

We also detected a monoallelic pathogenic variant of *SLC34A3* in an 18-year-old male who had borderline serum phosphorus, hypercalciuria, normal kidney function, and nephrocalcinosis. This patient’s clinical phenotype confirms that heterozygous patients can have manifestations of HHRH.

The genetic test also revealed one patient carrying a monoallelic variant c.(?_-1)_(*1_?)del, which is an entire deletion of the *CLCNKB* gene. Biallelic variants cause Bartter syndrome type 3. This is a 23-year-old male with a past medical history of urethral stone at the age of 5, followed by another two episodes of renal colic, with calcium oxalate and carbapatite stone composition. He was admitted to our clinic for a new episode of renal colic. Laboratory tests showed hypercalcemia, normokalemia, hypomagnesemia, metabolic alkalosis, and normal serum creatinine. He presented with a positive furosemide fludrocortisone test. The clinical diagnosis before the genetic test was dRTA.

#### 3.2.4. Monoallelic VUS

We detected monoallelic VUS in the *SLC3A1* gene (one patient), the *SLC22A12* gene (three patients), and the *SLC26A1* gene (one patient) (Table 5).

The monoallelic VUS in the *SLC3A1* gene was identified in a 39-year-old woman with bilateral severe medullary nephrocalcinosis, calcium, and phosphate containing kidney stones and CKD stage 2.

Our first patient was a 37-year-old female with recurrent NL, multiple ureteroscopy with lithotripsy interventions, urinary tract infections, and a family history of NL. The stone analysis showed a mixed composition: calcium phosphate, calcium oxalate, and struvite. At presentation, she had a borderline low uric acid level and normal serum creatinine. Genetic analysis revealed a heterozygous VUS missense mutation of *SLC22A12*. The second patient, a 35-year-old female, was admitted with kidney failure, urinary sepsis, and bilateral obstructive nephropathy, with grade V hydronephrosis due to staghorn stones. Her medical history included severe bilateral NL diagnosed at the age of 31 years and multiple urological interventions. She started hemodialysis and then a bilateral nephrectomy was performed, one after the other, within a month. Laboratory tests showed a normal uric acid level in the context of severe kidney failure. Genetic analysis showed a heterozygous VUS missense mutation in the *SLC22A12* gene. We underline that both patients were heterozygous carriers of the *SLC22A12* gene VUS mutation, and both developed severe NL. The third patient was a 28-year-old woman with NL, dysplastic kidney, numerous bilateral renal cysts, tubule–interstitial kidney disease, diabetes mellitus, hyperparathyroidism, and CKD G4. The genetic test showed a combination of two monoallelic VUS variants in *SLC22A12* and *HNF1B*. At referral, serum uric acid was normal, which could be explained by the severe kidney involvement and the *HNF1B* mutation-associated phenotype. We also identified an interesting association of two VUS in the *SLC26A1* and *HNF1B* genes in a patient with a clinical diagnosis of ADTKD, bilateral NL, and uric and oxalic diathesis.

## 4. Discussion

Nephrolithiasis and nephrocalcinosis are only symptoms, not a disease, and the role of the nephrologist is to diagnose the cause, which could be complex, including environmental, genetic, and metabolic factors. We retrospectively analyzed a single-center cohort of 49 patients with NL and/or NC for the presence of mutations in genes known to cause NL/NC. Overall, we found that 59.2% of patients had an abnormal variant identified, of which 28.6% had Mendelian diseases, 16.3% had P/LP monoallelic variants, and 14.3% had VUS. The percentage of Mendelian disease cases was remarkably high in our adult cohort. The high diagnostic yield could be explained by selection bias, as we used extremely selective inclusion criteria and patients were enrolled from a single center, which was also a tertiary care center. In addition, 7 of 14 patients diagnosed with Mendelian disease had a clinical diagnosis that was confirmed by genetic testing. Although 7 cases of Mendelian diseases were diagnosed by clinical phenotypes, the other 22 patients with positive genetic tests remained clinically undiagnosed and were considered to have idiopathic NL. Prior studies support the evidence that Mendelian (monogenic) diseases account for a significant proportion of kidney stone formers. Previous studies investigating the prevalence of genetic NL/NC in adult patients identified Mendelian diseases in 2.9–11.4% out of the studied cohorts with kidney stone disease [17,19,21,28]. The diagnostic yield increased to 29.4% in a recent study conducted on NL or NC patients with onset before the age of 25 [29]. Halbritter et al. investigated 116 adults and 106 children with NL or NC and showed that 11.4% of adult patients had a monogenic kidney stone disease and that in approximately 40% of cases the genetic diagnosis contributed to additional etiologic and diagnostic information and had management implications [17]. Anderegg et al. performed whole exome sequencing for 787 patients from the Bern Kidney Stone Registry who had at least one past kidney stone episode, as well as 114 non-kidney stone formers. They found a Mendelian disease among 2.9% of kidney stone formers and monoallelic variants predisposing them to NL among 8.1% of kidney stone formers [19]. A recent systematic review of the currently available literature included 13 studies on 1675 patients (23% adult populations) and found a diagnostic yield of 8% for the adult population [30].

In our cohort, NL associated with NC and the presence of CKD were the characteristics that significantly differentiated patients with positive genetic tests from those with negative genetic tests. Previous studies have shown that CKD and NC are suggestive features for genetic stone diseases [30]. Family history of NL/NC, recurrent NL, presence of bilateral NL, and age of onset of stone disease were not significantly different in patients with positive genetic tests compared with those in patients with negative genetic tests, probably due to our small sample size. Previous studies suggest that younger age of the onset of NL/NC and family history are factors correlated with the higher rates of molecular genetic diagnosis [17,29,30].

Among our patients, the disease was diagnosed late, in the advanced stages of CKD, in eight patients with positive genetic tests, including six patients with CKD G4 or G5 and two patients on renal replacement therapy (one patient on hemodialysis and one patient with a kidney transplant). Moreover, although 36.7% of the patients had NL and/or NC onset at a pediatric age, the molecular diagnosis was performed in adulthood. In our dRTA patients, we observed that early age of onset, nephrocalcinosis, and family history can help to differentiate patients with inherited dRTA from patients with other etiologies for dRTA. For the positive patients, family screening should be initiated for early diagnosis of other family members. The lack of recognition and awareness of genetic kidney stone disease, the variability of the phenotype, or changes in disease characteristics due to advanced kidney disease frequently resulted in delays in diagnosis and treatment. An example of phenotypic variability is the presence of calcium-based stones in a few cystinuria patients from our cohort. The monoallelic variants of the cystinuria genes with calcium-containing kidney stones were described in previous studies [16,17,31,32]. A high degree of clinical suspicion and a prompt diagnosis are essential to begin management approaches and potential treatments early in the disease course.

There is also increasing evidence regarding the impact of monoallelic variants. The identification of an abnormal monoallelic variant is considered a predisposing factor for NL/NC. The phenotype of patients with P/LP monoallelic predisposing factors can vary from a characteristic disease phenotype to milder phenotypes. In our group, 16.3% had P/LP monoallelic variants predisposing them to NL. Four cystinuria patients had a monoallelic P/LP pathogenic mutation, two in the *SLC3A1* and two in the *SLC7A9* genes, all of them presenting manifestations of cystinuria. Other comprehensive studies regarding the clinical and genetic analysis of patients with cystinuria showed that a single detectable mutation could explain the disease phenotype [33,34].

The combination of two risk factors was seen in two of our patients from the same family who had two different monoallelic mutations in the *AGXT* and *SLC34A3* genes. The clinical phenotype was not indicative of either PH1 or HHRH. Previous studies have suggested that patients heterozygous for *SLC34A3* pathogenic variants have a milder phenotype that includes borderline-low serum phosphate levels, hypercalciuria, and NC [35,36]. The presence of monoallelic AGXT and SLC34A3 variants together points to the contribution of multiple metabolic perturbations, such as oxalate and phosphate handling, which may converge on common pathological endpoints like mitochondrial dysfunction and apoptotic cell death. Apoptosis, characterized by oxidative stress, cytochrome c release, and caspase activation, has been implicated in renal tubular damage and is found to contribute to stone formation and nephrocalcinosis [37]. The literature data regarding the phenotype of patients with monoallelic variants of the *AGXT* gene is limited and does not mention the existence of both monoallelic *AGXT* and *SLC34A3* mutations simultaneously. Genetic diagnosis helped us to identify the predisposing factors for NL and to personalize their treatment.

In our cohort, *SLC34A3* abnormal variants were detected in four patients, being the second most common after cystinuria. Recent studies have shown the importance of monoallelic variants and their impact on the patients’ phenotypes. The study by Sadeghi-Alavijeh et al. provides evidence of clinical relevance for *SLC34A3* variants [38]. Rare monoallelic variants in *SLC34A3* are insufficient to cause Mendelian disease but have a higher disease risk [38]. Two investigations in cohorts of affected families indicated that heterozygous *SLC34A3* carriers could be symptomatic [35,36]. Monoallelic variants of other genes, such as *CYP24A1* and *SLC34A1,* have also been proven to have a role in increasing the risk of kidney stones. The proof comes from large genome-wide studies and clinical studies [27,39].

In our cohort, seven (14.3%) patients had VUS, one patient had biallelic VUS, and six patients had monoallelic VUS. Of the detected VUS, three were monoallelic variants in the *SLC22A12* gene. In terms of phenotype, two patients presented with severe NL and one of these patients with ESKD. Another patient had biallelic VUS mutations in *SLC34A3* with a suggestive phenotype for HHRH. We emphasize the importance of reporting these cases to generate additional evidence that could allow the reclassification of these variants. It is also important to report the new variants. In our study we found 2 new variants: c.690G>A, p.Trp230* in the *SLC7A9* gene, and c.4558G>A, p.Val1520Ile variant in the *ADCY10* gene. Until now, we do not have a follow-up testing for VUS variants. Also, a segregation analysis of the family members for the patient with VUS in the *ADCY10* gene could lead to the reclassification of the variant, if it is present in all members with NL, but absent in all other family members. Careful interpretation of VUS is necessary, based on the strength of published data for the gene and variant, clinical phenotype, and segregation in additional affected family members. In our center, we follow up with patients who had VUS and a highly suspected genetic etiology and recommend segregation analysis. During the monitorization we revisit existing literature and databases to see whether the VUS classification occurred. Interpreting VUS remains challenging and need further research.

Knowing the molecular diagnosis and the pathophysiology of the disease, a preventive treatment and a better management could be implemented [40]. The personalized management includes the following: initiating the specific treatment, avoiding vitamin D for patients with infantile hypercalcemia type 1, quantifying the urinary metabolites such as cysteine or oxalate to appreciate the response to hydration, to urinary alkalinization, and to the specific treatment and preventing the associated bone disease. An excellent example of personalized therapy after the molecular diagnosis is the patient with primary hyperoxaluria type 1, in whose case we initiated the newly introduced small interfering RNA treatment with a very good therapeutic response.

Genetic testing is also beneficial in cases of atypical clinical presentation or for patients whose features of the genetic disease are masked by advanced kidney impairment. Another important implication of the genetic diagnosis are the benefits of family screening and early diagnosis of other members of the family. Also, knowing the correct diagnosis leads to avoiding the disease recurrence after kidney transplant that could compromise the renal graft.

There are some limitations of our study, though. One of the most important limitations is the small size and the monocentric nature of the cohort. Our cohort reflects a high-risk subset of patients with NL and/or NC and not all stone former patients seen in our center. The use of strict inclusion criteria favors more severe cases, which might limit generalizability to broader populations. Given the small sample size of the cohort, the analysis could be underpowered especially for small effect sizes, especially for subgroups stratified by stone composition or genetics. In addition, the generalizability of our findings may be limited by the demographic, genetic and phenotypic characteristics of the study population. Also, the study was retrospective, and for some patients we missed detailed clinical and laboratory information, often due to the unavailability of certain tests. For example, data regarding the urinary metabolic assessment were available for only 12 (24.5%) of the patients, which was not sufficient for the statistical presentation of the data. Another limitation concerns the use of the nephrolithiasis gene panel and not the whole exome sequencing, which could limit novel gene discovery. Interpretation of the results must take this into account all of these aspects. Future studies with larger, multiethnic cohorts and integrative approaches combining genomics with metabolomics may help clarify the limitations of our study.

The strength of our study comes from the high yield of molecular diagnosis that helped us identify a large variety of causes of genetic NL and/or NC, diagnose an important number of cases, and therefore improve the disease management. Our study reported the experience of the largest Romanian cohort of adult patients with NL and/or NC evaluated through genetic testing.

## 5. Conclusions

This study showed an increased effectiveness of molecular diagnosis, with abnormal mutations in known nephrolithiasis/nephrocalcinosis genes identified in 59.2% of adult patients with NL and/or NC. We presented the phenotypes of the positive patients, with practical implications. Genetic testing should be considered in selected adult patients with NL and/or NC. In our cohort, NL associated with NC and the presence of CKD were the characteristics that differentiated the patients with positive genetic tests from those with negative genetic tests.

We underline the benefits of genetic testing in NL and/or NC, with results leading to accurate diagnosis, changes in disease management, and precision medicine, which could significantly influence the long-term outcomes. We conclude that molecular diagnosis improves patient management, prevents or delays chronic kidney disease, and offers the possibility of genetic counseling and family screening.

## Figures and Tables

**Table 1:** Characteristics of the study cohort according to the results of the diagnostic genetic test.

	Characteristics	Positive Genetic Test (N = 29)	Negative Genetic Test (N = 20)	*p* Value
General Features	Age at genetic test, years, mean ± SD	34.86 ± 9.23	35.50 ± 11.65	0.83
	Age at onset, years, mean ± SD	20.52 ± 9.68	24.20 ± 10.71	0.21
	Gender, *n* (%)			0.25
	Male	12 (41.4%)	11 (55%)	
	Female	17 (58.6%)	9 (45%)	
	Hypertension, *n* (%)	1 (3.4%)	5 (25%)	0.03
	Diabetes, *n* (%)	3 (10.3%)	0 (0%)	0.26
	Obesity (BMI ≥ 30 kg/m^2^), *n* (%)	2 (6.9%)	1 (5%)	1
	Dyslipidemia, *n* (%)	3 (10.3%)	1 (5%)	0.63
	Hyperuricemia, *n* (%)	5 (17.2%)	3 (15%)	0.83
	Family history of NL/NC, *n* (%)	20 (69%)	14 (70%)	0.93
	Nephrolithiasis, *n* (%)	18 (62.1%)	19 (95%)	0.10
	Recurrent NL, *n* (%)	23 (79.3%)	15 (75%)	0.75
	Bilateral NL, *n* (%)	22 (75.9%)	13 (65%)	0.45
	Nephrocalcinosis, *n* (%)	2 (6.9%)	1 (5%)	1
	NL + NC, *n* (%)	9 (31%)	0 (0%)	0.007
	CKD, *n* (%)			0.03
	G1	9 (31%)	8 (40%)	
	G2	5 (17.2%)	10 (50%)	
	G3	7 (24.1%)	1 (5%)	
	G4	4 (13.8%)	1 (5%)	
	G5 Dialysis/KT	2 (6.9%) 2 (6.9%)	0 (0%) 0 (0%)	
Blood Parameters	Total calcium, mg/dL, mean ± SD	9.36 ± 0.71	9.73 ± 0.70	0.10
	Ionized calcium, mg/dL, mean ± SD	4.04 ± 0.18	4.05 ± 0.15	0.83
	Phosphorus, mg/dL, mean ± SD	3.40 ± 0.76	3.20 ± 0.72	0.45
	Magnesium (xx) mean ± SD	2.00 ± 0.26	1.96 ± 0.18	0.59
	Uric acid, mg/dL, mean ± SD	5.88 ± 1.79	5.74 ± 1.25	0.76
	Intact parathormone, ng/L, median (IQR)	86.5 (47.5–132.9)	64.3 (55.6–93.8)	0.72
	eGFR, CKD-EPI no race 2021 equation, mL/min/1.73 m^2^, median (IQR)	69.7 (41.0–98.0)	85.5 (77.0–121.3)	0.03
	Composition			0.10
Stone Composition *	Calcium oxalate monohydrate, *n* (%)	7 (25.9%)	3 (15%)	
	Calcium oxalate dihydrate, *n* (%)	2 (7.4%)	2 (10%)	
	Uric acid, *n* (%)	2 (6.9%)	1 (5%)	
	Cystine, *n* (%)	3 (11.1%)	0 (0%)	
	Calcium phosphate, *n* (%)	7 (24.1%)	1 (5%)	
	Unknown, *n* (%)	5 (17.2%)	11 (55%)	
History of surgical interventions	Ureteroscopy with lithotripsy, *n* (%)	14 (48.3%)	9 (45%)	0.82
	ESWL, *n* (%)	7 (24.1%)	1 (5%)	0.11
	Percutaneous nephrolithotomy, *n* (%)	4 (13.8%)	3 (15%)	0.90
	Pyelolithotomy, *n* (%)	5 (17.2%)	1 (5%)	0.37
	Nephrectomy, *n* (%)	2 (6.9%)	0 (%)	1

N, number; SD, standard deviation; IQR, interquartile range; %, percentage; NL, nephrolithiasis; NC, nephrocalcinosis; NL + NC, nephrolithiasis plus nephrocalcinosis; CKD, chronic kidney disease; KT, kidney transplant; eGFR, CKD EPI, estimated glomerular filtration rate calculated with the Chronic Kidney Disease Epidemiology Collaboration; *, not available for 5 patients, 3 with positive diagnostic test and 2 with negative diagnostic test; ESWL, extracorporeal shock wave lithotripsy.

**Table 2:** Genotypes and phenotypes of patients diagnosed with Mendelian NL/NC.

Genetic Diagnosis	Patient No.	Sex/ Age (yrs)	Genetic Testing (Gene, Variant)	Inheritance	Allelic State	ACMG Class	On- Set Age (Yrs)	Clinical Diagnosis Before Genetic Test	Phenotype
Cystinuria type A	1	M/45	*SLC3A1,* c.1354C>T, p.Arg452Trp; c.1094G>T, p.Arg365Leu	AR	com het	P	35	Cystinuria	Cystine NL, CKD G5, unilateral renal cysts
	2	F/43	*SLC3A1*, c.1400T>C, p.Met437Thr	AR	hom	P	25	Cystinuria, polycystic kidney disease (PKD1 mutation c.4175_4176del, p.Val1392Alafs*38)	Cystine NL, CKD G3a, bilateral kidney cysts
Cystinuria type B	3	M/53	*SLC7A9,* c.313G>A, p.Gly105Arg; c.690G>A, p.Trp230*	AR	com het	P, LP	14	Cystinuria	Cystine NL, CKD G4
	4	F/52	*SLC7A9*, c.313G>A, p.Gly105Arg; c.217G>A, p.Gly73Arg	AR/ AD	com het	P, LP	29	ESKD, NL	NL, ESKD, hemodialysis
Autosomal dominant distal renal tubular acidosis	5	F/27	*SLC4A1*, c.1765C>T, p.Arg589Cys	AD/AR	het	P	18	dRTA	Carbapatite NL, medullary NC, dRTA, CKD G3a
	6	M/39	*SLC4A1*, c.1825G>A, p.Gly609Arg	AD	het	P	12	Medullary sponge kidney, dRTA	Carbapatite NL, medullary NC, dRTA, developmental delay, CKD stage G3a
	7	M/35	*SLC4A1*, c.1825G>A, p.Gly609Arg	AD	het	P	13	dRTA	Carbapatite NL, medullary NC, developmental delay dRTA, CKD G3b
Dent disease	8	M/53	*CLCN5*, c.1561C>T, p.Leu521Phe	XL	hem	LP	28	NL, NC Polycystic kidney disease	NL and NC, ESKD starting at age 28, receiving RRT (HD, afterwards KT)
	9	F/37	*CLCN5*, c.794G>A, p.Ser265Asn	XL	het	LP	37	NC	Severe medullary NC, mild proteinuria, CKD G1
Familial hypomagnesemia with hypercalciuria and nephrocalcinosis	10	F/20	*CLDN16* c.646C>T, p.Arg216Cys	AR	hom	P	7	NL, NC, tubulo-interstitial disease	NL and NC, hypomagnesemia, mild hypocalcemia, hypophosphatemia, hypercalcemia, proteinuria, CKD G4
Infantile hypercalcemia type 1	11	M/25	*CYP24A1*, c.428_430del, p.Glu143del; c.443T>C, p.Leu148Pro	AR	com het	P	24	NC	Hypercalcemia, low iPTH, bilateral NC, CKD G2, right kidney cyst
Primary hyperoxaluria type 1	12	F/42	*AGXT*, c.33del, p.Lys12Argfs*34; c.508G>A, p.Gly170Arg	AR	com het	P	6	PH1, NL, NC	Calcium oxalate monohydrate NL, increased urinary and plasma oxalate, medullary NC, CKD G4
Bartter syndrome type 2	13	F/32	*KCNJ1*, c.658C>T, p. Leu220Phe	AR	hom	P	12	NC, NL, type 3 RTA, diabetes insipidus, hyperreninemic hyperaldosteronism	NL, NC, hypokalemia, hypercalciuria, weakness, polyuria, polydipsia, hyperreninemic hyperaldosteronism, CKD G3a, parathyroid adenoma
Autosomal dominant tubulointerstitial kidney disease	14	F/36	*UMOD*, c.686>A, p.(Met229Lys)	AD	het	P	28	ADTKD, NL	Progressive CKD, bland urinalysis, hyperuricemia, sporadic kidney stones, CKD G2

M, male; F, female; AD, autosomal dominant; AR, autosomal recessive; hem, hemizygous; het, heterozygous; hom, homozygous; com het, compound heterozygous; CKD, chronic kidney disease; NL, nephrolithiasis; NC, nephrocalcinosis; dRTA, distal renal tubular acidosis; ESKD, end-stage kidney disease; RRT, renal replacement therapy; HD, hemodialysis; KT, kidney transplant; iPTH, intact parathyroid hormone.

**Table 3:** Genotypes and phenotypes of patients with possible genetic diagnosis of NL/NC.

Possible Genetic Diagnosis	Patient No.	Sex/ Age (Yrs)	Genetic Testing	Inheritance	Allelic State	ACMG Class	Onset Age (Yrs)	Clinical Diagnosis Before Genetic Test	Phenotype
Autosomal dominant familial idiopathic hypercalciuria	1	F/31	*ADCY10*, c.4558G>A, p.Val1520Ile	AD	Het	VUS	15	PH, NL	NL with calcium oxalate monohydrate stone composition and normal kidney function
Hereditary hypophosphatemic rickets with hypercalciuria (HHRH)	2	M/40	*SLC34A3*, c.274G>A, p.Val92Ile; c.286G>A, p.Ala96Thr	AR	com het	VUS	37	Idiopathic NL	Recurrent NL with multiple surgical interventions, hypophosphatemia, and normal kidney function

M, male; F, female; AD, autosomal dominant; AR, autosomal recessive; het, heterozygous; com het, compound heterozygous; VUS, variant of uncertain significance; PH, primary hyperoxaluria; NL, nephrolithiasis.

**Table 4:** Genotypes and phenotypes of patients diagnosed with P/LP monoallelic variants as predisposing factors for NL/NC.

Patient No.	Sex/ Age (Yrs)	Genetic Testing (Gene, Variant)	Inheritance	Allelic State	ACMG Class	Onset Age (Yrs)	Clinical Diagnosis Before Genetic Test	Phenotype
1	F/32	*SLC3A1*, c.1400T>C, p.Met467Thr	AR	het	P	30	NL	Calcium carbonate and struvite NL, normal kidney function, many episodes of renal colic (~20/year) with multiple urological interventions
2	M/30	*SLC3A1*, c.(891+1_892-1)_(1617+1_1618-1)dup	AR	het	P	28	NL	Calcium oxalate dihydrate NL, normal kidney function
3	M/26	*SLC7A9*, c.217G>A, p.Gly73Arg	AD	het	LP	25	NL	Calcium oxalate NL, normal kidney function
4	F/30	*SLC7A9*, c.313G>A, p.Gly105Arg	AD	het	P	20	NL	Calcium oxalate NL, normal kidney function
5	F/28	*AGXT*, c.107G>A, p.Arg36His and *SLC34A3,* c.575C>T, p.Ser192Leu	AR	het	P	18	NL	Calcium oxalate monohydrate NL, slight increase in urinary oxalate, CKD G2
6	F/25	*AGXT,* c.107G>A, p.Arg36His and *SLC34A3*, c.575C>T, p.Ser192Leu	AR	het	P	20	NL	Calcium oxalate monohydrate NL, slight increase in urinary oxalate, CKD G2
7	M/18	*SLC34A3,* c.1304del, p.Ser435Thrfs*46	AR	het	P	17	NC	Bilateral medullary NC, borderline phosphorus, metabolic alkalosis, hypercalciuria, normal kidney function
8	M/23	*CLCNKB*, c.(?_-1)_(*1_?)del, p.0	AR	het	P	5	dRTA	NL with calcium oxalate and carbapatite stone composition, dRTA, normal kidney function

M, male; F, female; AD, autosomal dominant; AR, autosomal recessive; het, heterozygous; CKD, chronic kidney disease; CKD, chronic kidney disease; NL, nephrolithiasis; NC, nephrocalcinosis; dRTA, distal renal tubular acidosis.

**Table 5:** Genotypes and phenotypse of patients diagnosed with monoallelic VUS.

Patient No.	Sex/ Age (Yrs)	Genetic Testing	Inheritance	Allelic State	ACMG Class	On- Set Age (Yrs)	Clinical Diagnosis Before Genetic Test	Phenotype
1	F/39	*SLC3A1* c.1684G>C, p.Glu562Gln	AR	het	VUS	33	NL, NC	Oxalate and phosphate NL, bilateral severe medullary NC, CKD G2
2	F/37	*SLC22A12*, c.412G>A, p.Val138Met	AR	het	VUS	29	NL	Calcium phosphate and struvite NL, multiple urological interventions, hypouricemia, normal kidney function
3	F/35	*SLC22A12*, c.431T>C, p.Leu144Pro	AR	het	VUS	31	NL, ESKD	Staghorn NL with obstructive nephropathy, ESKD
4	F/28	*SLC22A12*, c.1427C>A, p.Ala476Asp, and *HNF1B* c.544 + 5G>A, het, VUS	AR	het	VUS	11	Genetic tubulo- interstitial disease, CKD G4	NL, dysplastic kidney, renal cysts, tubule–interstitial kidney disease, diabetes mellitus, hyperparathyroidism, CKD G4
5	M/42	*SLC26A1* c.2007C>G, p.Asp669Glu and *HNF1B* c.867C>G, p.Asn289Lys, het, VUS	AR	het	VUS	4	NL, ADTKD	Bilateral NL, uric and oxalic diathesis, multicystic and dysplastic kidney, tubulointerstitial kidney disease, CKD G3a, diabetes mellitus, pancreatic hypoplasia

M, male; F, female; AR, autosomal recessive; het, heterozygous; VUS, variant of uncertain significance; NL, nephrolithiasis; NC, nephrocalcinosis; CKD, chronic kidney disease; ESKD, end-stage kidney disease; ADTKD, autosomal dominant tubulointerstitial kidney disease.

## Data Availability

The data presented in this study are available upon request from the corresponding author due to ethical reasons.
